# Characterization of Ceria Nanoparticles as Abrasives Applied with Defoaming Polymers for CMP (Chemical Mechanical Polishing) Applications

**DOI:** 10.3390/polym16060844

**Published:** 2024-03-19

**Authors:** Sohee Hwang, Woonjung Kim

**Affiliations:** Department of Chemistry, University of Hannam, Daejeon 34430, Republic of Korea; naturalj.sohee@gmail.com

**Keywords:** chemical mechanical polishing/planarization (CMP), ceria nanoparticle, dispersant, defoaming polymer, polishing selection ratio

## Abstract

Chemical mechanical polishing/planarization (CMP) is an essential manufacturing process in semiconductor technologies. This method combines chemical and mechanical forces to smooth the surfaces of wafers. The effectiveness of CMP relies on a carefully chosen slurry, demanding a sophisticated manufacturing technology. This technology must seamlessly integrate both chemical composition and mechanical elements, highlighting the intricate synergy required for successful semiconductor fabrication. Particularly in milling processes, if agglomerated particles due to slurry particle corrosion are present during polishing, uneven polishing, numerous fine scratches occur, leading to an increase in roughness and a deterioration in the quality of the finished surface. In this study, to overcome the issue of particle agglomeration and uneven polishing in commonly used ceria nanoparticle slurries during CMP processes, we investigated the ceria nanoparticle behavior based on styrene–maleic acid (SMA) dispersant polymer applied with three types of defoaming polymers. The investigations are expected to open up the possibility of utilizing ceria nanoparticles with applied defoaming polymer as an abrasive for advanced CMP applications. All samples were characterized by DLS (dynamic light scattering), SEM-EDX (scanning electron microscopy–energy dispersive X-ray spectroscopy), pH, conductivity, viscosity, a 10-day stability test at 60 °C, the AF4 test, and the polishing rate efficiency test. Our research demonstrates a significant improvement achieved through the use of SMA dispersant polymer, resulting in a polishing selection ratio exceeding 80 for oxide and nitride films. The G-336 defoaming polymer utilized here is expected to serve as a viable alternative in CMP processes by providing stable uniformity.

## 1. Introduction

Chemical mechanical polishing/planarization (CMP) is a universally recognized procedure for providing a global surface finish to different materials for a variety of applications such as jewelry, precise optics, laser techniques, and electronics [1,2,3,4]. It is a surface smoothening process with the combination of controlled mechanical force such as pressure or the relative motion of the polishing tool and a chemical reaction for achieving desired materials removal in the semiconductor industry [1,4,5,6,7,8]. 

In general, the advantages of using CMP as a global planarization technique can be invalidated by contamination slurry chemicals and particles from abrasive particles, pattern-related defects like dishing and erosion, delamination, etc. Therefore, it is crucial to assess the requirements of a high oxide removal rate (RR) to overcome these new challenges for the CMP process.

**Figure 1:**
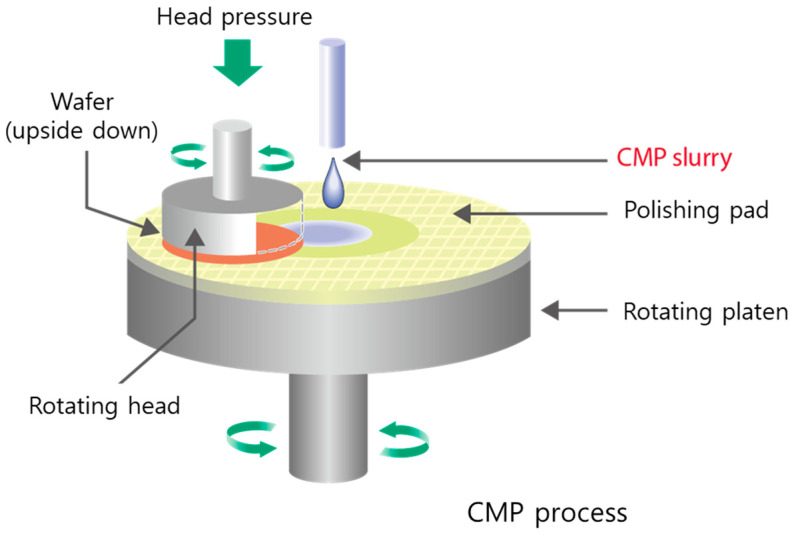
Schematic illustration of the CMP process using ceria nanoparticles as an abrasive.

CMP is a particularly important part of the integrated circuit (IC) manufacturing process. CMP treatment of the wafer surface can greatly improve the flatness of the wafer, which will affect the subsequent process. The process of polishing the fused silica surface with abrasives and the scratches caused by abrasives are shown in Figure 1 [9]. The fused silica adhered to the polishing head is placed on the polishing pad under a certain pressure, and the polishing head rotates at a constant speed while the polishing slurry drops on the polishing pad at a certain speed. In this process, the abrasive will leave certain scratches on the fused silica, which should be avoided as much as possible during the polishing process. The shape and hardness of the abrasive play a decisive role in the removal effect and scratch state of the material. Therefore, it is very important to find an appropriate abrasive shape to improve the polishing performance. The polishing rate varies depending on the size and concentration of the abrasive particles, so achieving the optimal particle size distribution (PSD) is crucial for maximizing RR without damaging the surface.

**Figure 2:**
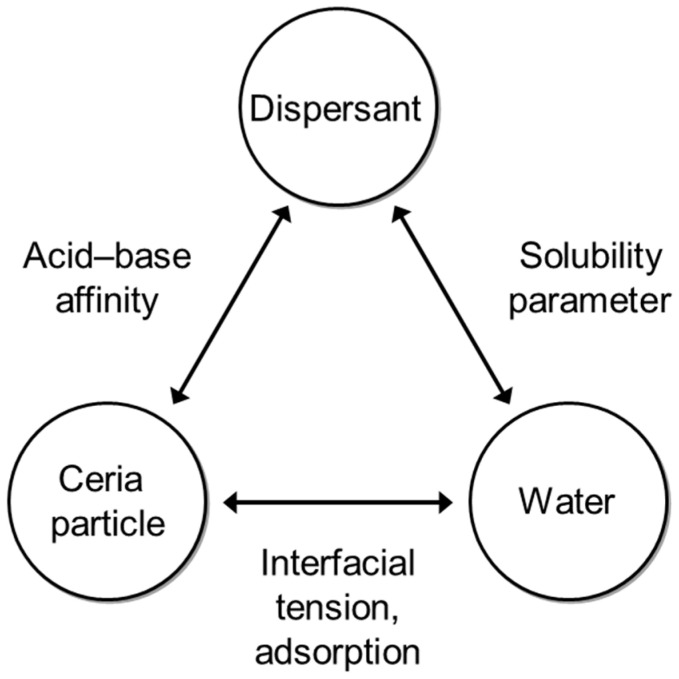
Importance of three components in ceria nanoparticle slurry.

Furthermore, achieving a balance of three components such as ceramic particles, water, and dispersants is crucial for the dispersion of colloidal slurries. Specifically, the dispersibility of ceramic particles within the slurry is significantly influenced by the concentration of dispersants, and variations in dispersant content can lead to particle agglomeration. Therefore, it is highly important to maintain a balance among the three components of ceramic particles, dispersants, and water in the composition of the slurry, as illustrated in Figure 2 [10,11,12].

Ceria-based polishing powders are typically produced by subjecting suitable precursors to thermal decomposition. These precursors commonly include cerium oxalates, hydroxides, acetates, and carbonates [13,14,15,16,17]. Cerium oxide (CeO_2_) is recognized for its elevated oxide removal rate (RR) owing to its strong chemical interaction with an oxide surface [18,19]. Various mechanisms explaining this interaction have been proposed by numerous researchers, with a consensus that the active sites for the reaction involve Ce^3+^ ions on the ceria surfaces [19,20]. The formation of an oxygen vacancy results in the reduction of cerium ions within the lattice, transitioning from Ce^4+^ to Ce^3+^. Ce^3+^ plays a pivotal role in initiating the reaction with the oxide surface forming strong Ce-O-Si chemical bonds. This strong adhesion accelerates the generation of Ce-O-Si bonds, subsequently enhancing the RR of SiO_2_ and exhibiting good selectivity for Si_3_N_4_ [19,20,21,22,23,24]. 

Studies have concentrated on enhancing the surface concentration of Ce^3+^ ions, leading to an elevated removal rate (RR) of the SiO_2_ layer. This is attributed to the strong interaction between ceria and SiO_2_ [19,23]. Kim’s group introduced a colloidal ceria abrasive featuring both spherical and nanocluster structures, characterized by a higher concentration of Ce^3+^ [19]. To enhance the oxide removal efficiency, several investigations have focused on increasing the concentration of Ce^3+^ ions in ceria abrasives. Kim et al. explored the impact of Ce^3+^ ion concentration on the removal rate (RR) of SiO_2_ layers and proposed a method for synthesizing ceria particles with a high concentration of Ce^3+^ ions by reducing the primary particle size. They reported that smaller particles demonstrate increased polishing efficiency attributed to the abundance of Ce ions [23].

Generally, the preparation methods of cerium oxide include calcination, the sol-gel method, the precipitation method, the hydrothermal method, the solvothermal method, the hydroxide-mediated method, etc., of which the calcination method is the most commonly used [25,26,27,28,29,30]. Indeed, calcined ceria slurry is anticipated to serve as a viable alternative slurry candidate, primarily due to the small size and regular shape of its particles in comparison with traditional calcined ceria particles [31]. 

**Figure 3:**
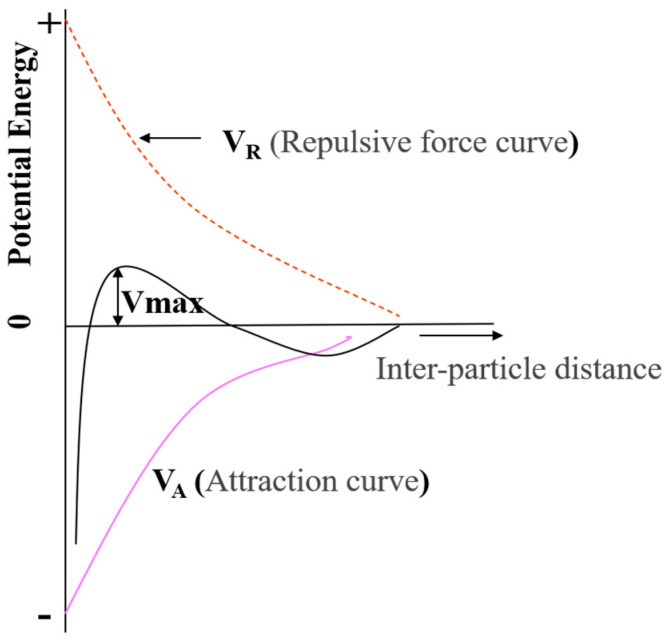
Schematic interaction energy vs. distance profiles of DLVO interaction. The attractive van der Waals and the repulsive electrostatic potentials form the total interaction energy.

According to DLVO theory [32], an appropriate dispersant concentration can regulate electrostatic double-layer interactions, preventing close contact between ceria nanoparticles and maintaining dispersion, thereby reducing cohesion. Furthermore, as the cohesion between particles decreases, scratches are minimized during the CMP process, allowing for more effective surface treatment and potentially increasing polishing rates and selectivity (as shown in Figure 3).

However, during the milling process, agglomerated particles resulting from slurry particle corrosion can lead to numerous fine scratches and an increase in surface roughness, thereby degrading the quality of the finished surface. Several studies have identified more suitable methods for surface roughness analysis, particularly in milling processes, when the presence of undifferentiated particles during polishing can lead to surface quality degradation and rough outcomes, potentially resulting in fine scratches [33,34]. 

To solve these problems and enhance the dispersion of calcined ceria, dispersants and surfactants are added to minimize inter-particle interactions. Several researchers have specifically investigated enhancing the dispersibility of ceria slurries using copolymers, and anionic polymers [35,36,37]. Dispersant-adjusted ceria particles are known to undergo transitions of bridging agglomeration-stable-flocculation depending on their physicochemical conditions such as pH and concentration [38].

**Figure 4:**
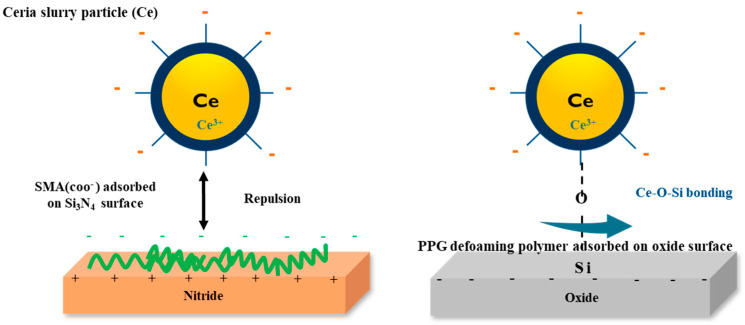
Schematic representation of SMA dispersant as an additive adsorbed in the ceria surface on nitride and oxide, respectively.

The application of a defoaming polymer is particularly beneficial. It helps prevent the formation of bubbles on the oxide surface, thereby enhancing the Ce-O-Si bonding force and resulting in a higher oxide polishing rate. By adding poly propylene glycol (PPG) defoaming polymer, it is possible to prevent bubbles on the oxide surface and increase the Ce-O-Si bonding force, resulting in a high oxide polishing rate. The nitride polishing rate can be reduced by suppressing the hydrolysis reaction of the nitride surface due to the strong bond of SMA to the ceria surface. Therefore, the selection ratio between oxide and nitride can be increased, as shown in Figure 4.

In this study, our objective is to develop a highly efficient CMP slurry. This slurry will incorporate calcined ceria nanoparticles along with an SMA dispersant polymer, with the addition of three types of defoaming polymers (PPG) using various concentrations based on the SMA dispersant in the previous work [39]. The goal is to overcome particle agglomeration, stable polishing uniformity, and scratches among particles. Additionally, we aim to enhance the polishing rate efficiency of the slurry through the use of these additives.

## 2. Experimental Methods

### 2.1. Preparation of Calcined Ceria Nanoparticles

Cerium carbonate hydrate (Ce_2_(CO_3_)_3_)∙6H_2_O powder was calcined at temperatures ranging from 500 to 1000 °C to obtain cerium oxide powder for use as abrasive particles in the polishing test. For the slurry preparation, deionized water (aquapuri 5 series by YOUNG IN SCIENTIFIC.Co., Ltd., Seoul, Republic of Korea ) and an acrylic acid-based dispersant polymer (Vanderbilt Minerals, LCC, Gouverneur, NY, USA) were utilized.

### 2.2. Preparation of the Ceria Slurry 

To manufacture the ceria slurry, 600 g of slurry, consisting of ceria powder (180 g), 27 g of dispersant polymer, and 393 g of distilled water, was processed using a Basket-mill (Tedi, JS Basket-mill Mill, Daejeon, Republic of Korea). During dispersion, beads with a size of 0.2 mm were utilized with a bead filling ratio set at 60%. Additionally, the milling process was conducted at 1500 rpm for 3 h. The obtained slurry was then diluted to achieve solid content with a fixed amount.

### 2.3. Polishing Experiments

Polishing tests were carried out using AP-300 equipment (CTS Company, Cheongju, Republic of Korea). Pads were set to rotate at a speed of 93 rpm under a downward load, and the slurry flow rate was maintained at 180 mL/min. A uniform polishing time of 60 s was set, and conditioning was performed for 10 min using a conditioner. The wafers used included plasma-enhanced tetraethylorthosilicate (PETEOS), silicon nitride, and Polysilicon. In addition, for the purpose of comparison, the pad before polishing was employed as a control group to evaluate the polishing rate for each slurry.

### 2.4. Characterizations 

To evaluate the stability of the manufactured slurry, we employed a pH meter (Thermo-scientific, OrionstarsA215, Waltham, MA, USA), conductivity meter (Thermo-scientific, OrionstarsA215 USA), and viscometer (Brookfield, DV Next Cone/Plate Rheometer, New York, NY, USA). Dynamic light scattering (DLS, ELS-2000, Otsuka Electronics, Japan) was used for measuring particle size and size distribution. On the other hand, AF (Asymmetrical Flow Field-Flow Fractionation) analysis was performed under the conditions of a flow rate of 0.6 mL/min, a cross-flow rate of 0.5 mL/min, and a carrier liquid solution consisting of 0.1% FL70^TM^ (Fisher chemical, detergent) and 0.01% NaN_3_ for size distribution analysis. For characterizing the shape and size of the ceria slurry particles, a field-emission scanning electron microscope (FE-SEM, JEOL-7800F, JEOL Ltd., Tokyo, Japan) was employed.

## 3. Results and Discussion

### 3.1. Preparation of Calcined Ceria Nanoparticles

**Figure 5:**
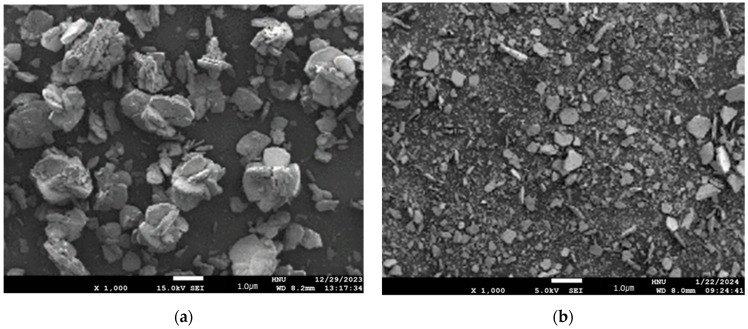
SEM images of cerium carbonate (**a**) and cerium oxide after calcined at 800 °C (**b**) (×1000).

Figure 5 shows SEM images of cerium carbonate (Ce_2_(CO_3_)_3_)∙6H_2_O and calcined ceria nanoparticles (CeO_2_). The cerium carbonate appears to have a size in the sub-micrometer range and exhibits significant agglomeration. On the other hand, the calcined ceria nanoparticles have sizes ranging from approximately tens to hundreds of nanometers and do not exhibit observable agglomeration. These changes in nanoparticle morphology and size can impact CMP performance. Nanoparticles with uniform size and shape can provide more consistent surface properties during the polishing process, thereby enhancing surface uniformity and finishing quality. This suggests the potential suitability of using them as abrasives for CMP slurry. 

The EDX results in Table 1 reveal a relatively consistent surface distribution of cerium (Ce), consistent with the analysis that confirms an increase in Ce content of over 70% after calcination at 800 °C. This suggests that the calcination pre-treatment conditions were well executed.

### 3.2. Properties of Synthesized SMA-1000 Dispersion Material

**Figure 6:**
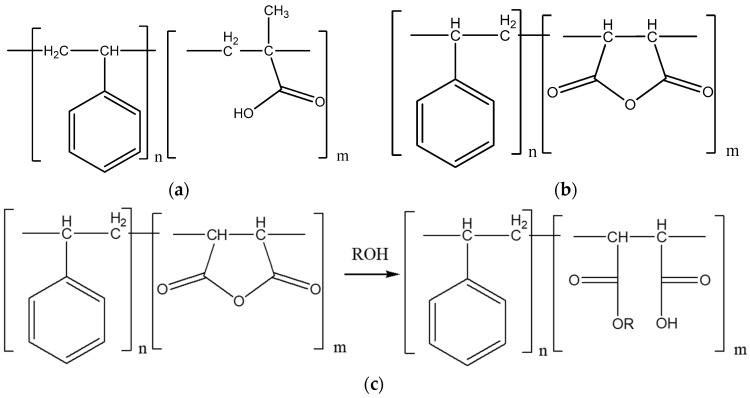
Chemical structure of the dispersant polymer of styrene acrylate series (SAA) (**a**), the styrene–maleic series (SMA) (**b**), and the synthetic procedure of the SMA dispersant polymer (**c**).

SMA (styrene–maleic acid) is a copolymer compound derived from the polymerization of styrene and maleic anhydride. A modified SMA dispersant polymer with vinyl functionality was synthesized using SMA monomers through radical reactions under alkaline conditions, as shown in Figure 6. The synthesized SMA dispersant polymer has a pH of 8.07 and a weight-average molecular weight (MW) of 8.60 × 10^4^, as listed in Table 2. It is believed that the stability of ceria nanoparticles could be improved when the pH of the modified SMA dispersant polymer is mildly alkaline, such as 8.07. The molecular weight of the modified SMA dispersant polymer can influence the particle size distribution within the CMP slurry. A higher molecular weight maintains a more stable dispersion, preventing the aggregation of ceria nanoparticles and maintaining dispersion. This improvement in dispersion enhances polishing efficiency in the CMP process.

**Figure 7:**
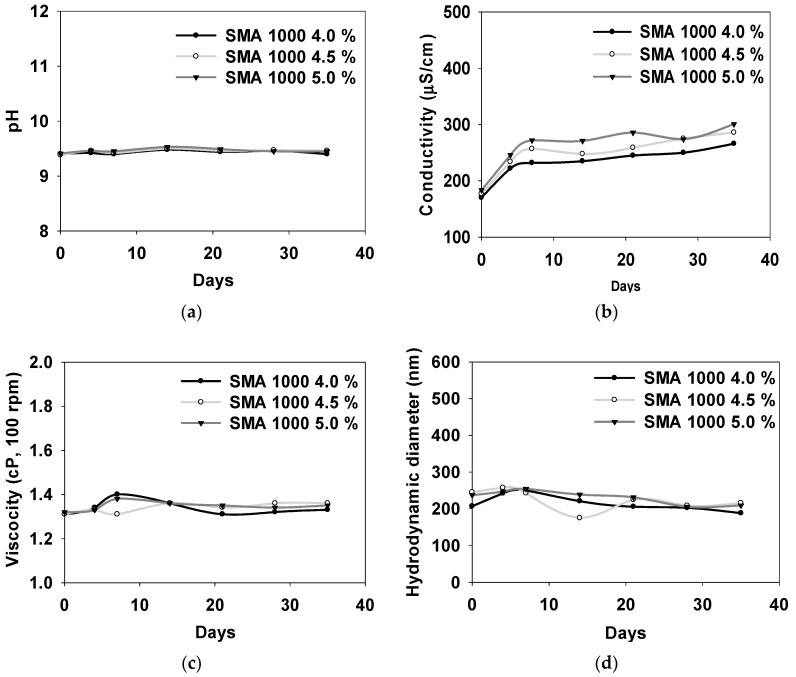
Stability test measured at 60 °C for 35 days after the addition of SMA-1000 dispersion; pH (**a**), conductivity (**b**), viscosity (**c**), and hydrodynamic diameter by DLS (**d**).

Figure 7 shows the stability tests of the SMA-1000 dispersant polymer at concentrations of 4.0%, 4.5%, and 5.0%, respectively. To determine the stability, the prepared dispersants were maintained at 60 °C for 35 days. Subsequently, the prepared SMA-1000 dispersant polymer was measured for pH, conductivity, viscosity, and particle size analysis. The pH, conductivity, and particle size increased from 9.43 to 9.46, 231 μS/cm to 262 μS/cm, and 216 nm to 232 nm, respectively. Meanwhile, the viscosity showed a slight increase from 1.34 cP to 1.35 cP with the rising concentration of the SMA-1000 dispersion material at concentrations of 4.0%, 4.5%, and 5.0%. These results are indicated in Table 3.

### 3.3. Properties of SMA-1000 Dispersion Applied with Three Types of Defoaming Polymers as Additives

Typically, ceria slurry with added SMA dispersant exhibits a satisfactory grinding efficiency. However, extended milling time is necessary to eliminate bubbles, which resulted in challenges for process application in our previous study. 

According to our goals, we aimed to find the optimal defoaming polymer among Depol, BYK, and G-336 by applying them with the modified SMA-1000 dispersant polymer, ensuring good compatibility and improving thermal stability, dispersion stability, and grinding efficiency.

**Figure 8:**
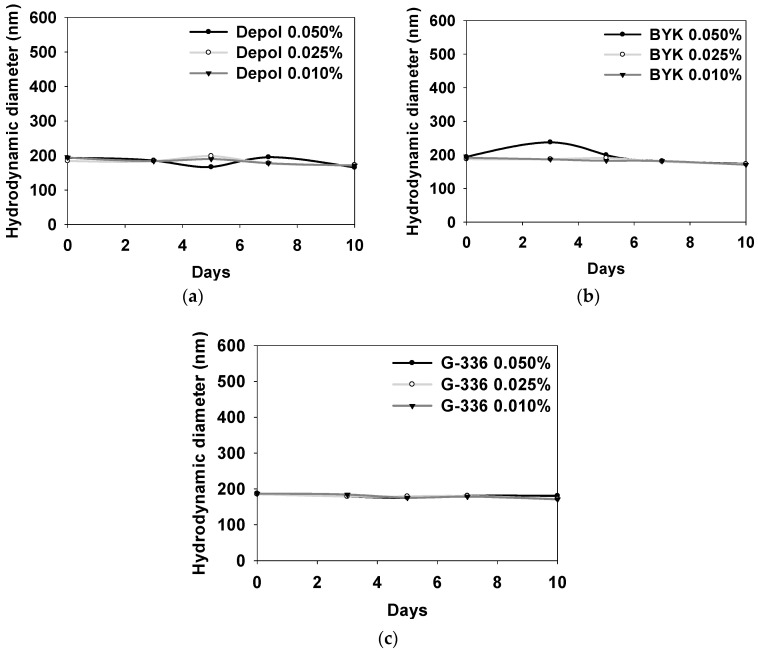
DLS results for stability according to 3 types of defoaming polymers with various concentrations at 60 °C for 10 days: Depol (**a**), BYK (**b**), and G-336 (**c**).

Figure 8 illustrates the DLS evaluation results for stability based on the defoaming polymer at various concentrations of 0.010%, 0.025%, and 0.050% at 60 °C over a 10-day period for Depol, BYK, and G-336 samples. Slight changes with increasing concentration are observed in particle size for the Depol and BYK defoaming polymers, while the G-336 defoaming polymer shows no significant variations in the DLS results as the concentration increases. The phenomenon of the G-336 defoaming polymer is attributed to its relatively higher stability compared with the other defoaming polymers. When the concentration of the defoaming polymer is 0.025% or less, both the defoaming efficiency and properties exhibit stable results in terms of storage stability (10 days at 60 °C).

Table 4 summarizes the stability tests of ceria slurry with the application of Depol, BYK, and G-336 deforming polymers as additives. As the concentration of the Depol defoaming polymer increased, the pH of the ceria slurry was measured at 9.68, 9.68, and 9.67, respectively. The conductivity was confirmed to be 205 μS/cm, 208 μS/cm, and 209 μS/cm, respectively.

There was no significant change in particle size measured at 183, 183, and 181 nm, respectively, corresponding to the viscosity at 1.33, 1.32, and 1.32 in the Depol deformer. In the case of an increasing concentration of the BYK defoaming polymer, the pH of the ceria slurry was measured at 9.68 in all samples. The conductivity was determined to be 206 μS/cm, 209 μS/cm, and 212 μS/cm, respectively. The viscosity was measured at 1.32 cP, 1.31 cP, and 1.31 cP, and the particle size was measured at 183 nm, 184 nm, and 197 nm, respectively. 

Finally, the pH showed a slight variation, measuring 9.64, 9.65, and 9.66, respectively with the G-336 defoaming polymer as the concentration increased. The viscosity remained nearly constant at 1.32 cP. On the other hand, the conductivity increased with concentration, reaching 218 μS/cm, 219 μS/cm, and 245 μS/cm, especially showing high conductivity with the G-336 defoaming polymer at a concentration of 0.05%. The particle size remained relatively stable, measuring 179 nm, 179 nm, and 180 nm, respectively. Considering these results, it is anticipated that the G-336 defoaming polymer, due to its high compatibility with the SMA-1000 dispersant polymer, will demonstrate effective grinding efficiency in the ceria slurry. 

### 3.4. Properties of Ceria Slurry Applied with Three Types of Defoaming Polymers as Additives 

**Figure 9:**
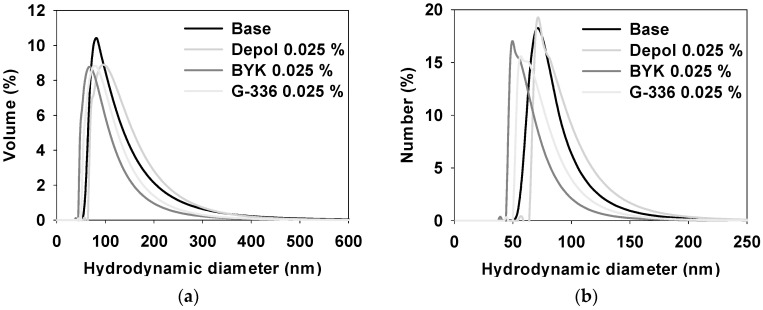
Particle size distribution (PSD) based on volume (**a**) and number (**b**) of ceria slurry by DLS analysis.

Figure 9 characterizes the particle size distribution (PSD) of a ceria slurry, comparing samples without any defoaming polymer (Base) to those with the addition of three different defoaming polymers (Depol, BYK, and G-336). The analysis is conducted in terms of both volume and number using DLS analysis. In both distributions, the sizes follow the order of Depol, Base, G-336, and BYK, showing a decrease in size. 

These findings suggest that the BYK and G-336 defoaming polymers might offer enhanced dispersibility and compatibility with the ceria slurry compared with Depol and Base. Detailed evaluation results can be found in Table 5.

**Figure 10:**
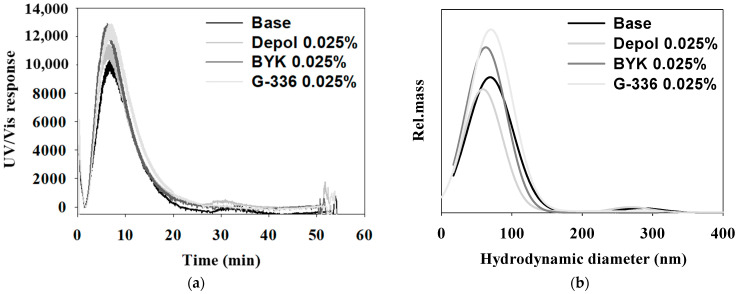
The AF4 (Asymmetrical Flow Field-Flow Fractionation) fractograms of ceria slurry obtained under varying conditions, including a base condition and with the addition of three different types of defoaming polymers. The fractograms (**a**) and size distribution (**b**) were measured as part of the analysis. Specific parameters for the AF4 setup included a channel flow rate of 0.6 mL/min and a cross-flow rate of 0.5 mL/min, and the carrier liquid was composed of water containing 0.1% FL-70 and 0.01% NaN_3_.

To obtain a more detailed size distribution with the addition of specific defoaming polymers, we conducted AF analysis on ceria slurries without a defoaming polymer (Base) and with three types of defoaming polymers at a concentration of 0.025%, as shown in Figure 10. The retention times were similar for all samples, and the main peak sizes were observed as follows: 72.2 nm for the sample without defoaming polymer, 62.6 nm for the Depol defoaming polymer, 73.3 nm for the BYK defoaming polymer, and 82 nm for the G-336 defoaming polymer. While the sizes analyzed with the BYK and G-336 defoaming polymers were smaller (in the previous DLS analysis) in the AF analysis, they were measured slightly larger compared with the Base and Depol samples. However, since no minor peaks were observed, this indicates a more uniform size distribution. A summary of the results of the AF analysis with the application of defoaming polymers is provided in Table 6.

**Figure 11:**
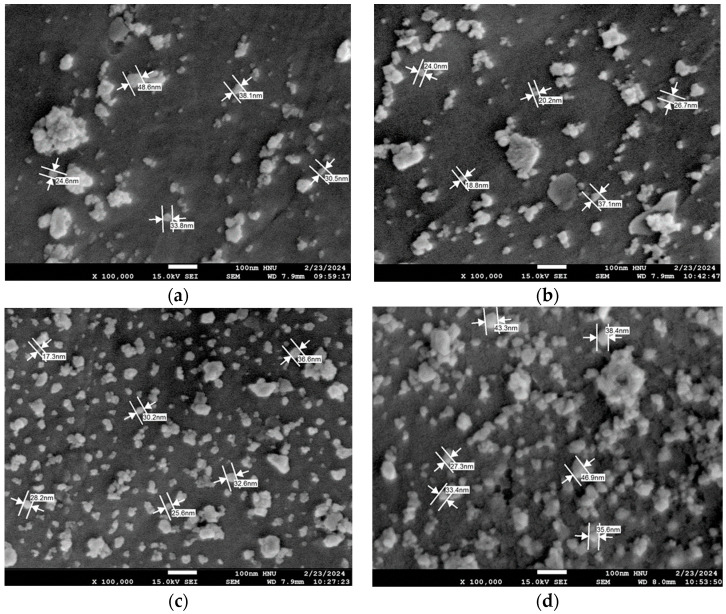
SEM image of ceria nanoparticles according to 3 types of defoaming polymers (×100,000): Base (**a**), Depol (**b**), BYK (**c**), and G-336 (**d**).

Figure 11 presents SEM images of the results of observing ceria nanoparticle sizes in ceria slurries without a defoaming polymer (Base) and with the application of Depol, BYK, and G-336 defoaming polymers using FE-SEM analysis. In the case of the Base and Depol defoaming polymer-applied slurries, ceria nanoparticles exhibit an irregular size distribution with a limited number of particles visible at a certain angle. In contrast, the slurries adapted with BYK and G-336 defoaming polymers show ceria nanoparticles with sizes ranging between about ca. 20 and 30 nm, and a significantly larger number of particles is observed. Based on the SEM results, it can be suggested that ceria nanoparticles with a consistent size in the range of approximately 20 to 30 nm, observed in the ceria slurries with BYK and G-336 defoaming polymers, may yield more impactful results in future polishing rate tests.

Furthermore, there was no apparent difference in the morphology of the ceria nanoparticles between the Base and the Depol defoaming polymer samples. However, distinctive aggregation of several hundred nanometers was observed in pure ceria particles (Base), while the modified ceria nanoparticles exhibited relatively good dispersibility. This further indicates the enhanced dispersibility of the surface-modified ceria particles. This can also be observed by examining the images before and after the addition of the G-336 defoaming polymer, as shown in Figure 11.

### 3.5. Polishing Test of Ceria Slurry Applied with Defoaming Polymers as Additives

Polishing rate experiments were carried out on thermally grown silicon oxide and nitride films on silicon wafers (Noel Technologies, Campbell, CA, USA) and were polished on a CTS’s company polisher using AP-300 Groove pads (Cheongju, Republic of Korea) made of polyurethane (IC 1010). Before each experiment, the polishing pad was conditioned for 1 min with a diamond grit conditioner using deionized water. The aqueous polishing slurry (1 wt%) was sonicated for 30 min and was placed on a roller miller to maintain good dispersion. The results of the CMP performance as a function of the applied three types of defoaming polymers for ceria slurry are shown in Table 7. 

**Figure 12:**
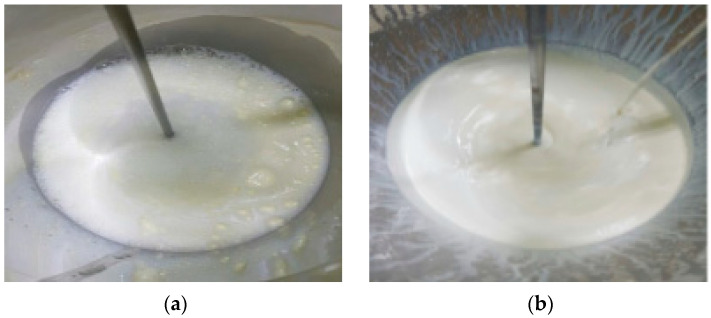
Photograph of ceria slurry before (**a**) and after the addition of the G-336 defoaming polymer (**b**).

The polishing rate efficiency of PETEOS for the ceria slurry samples, including the base sample without a defoaming polymer (Base) and those with Depol, BYK, and G-336 defoaming polymers, was measured at 3493 Å/min, 4650 Å/min, 5558 Å/min, and 5417 Å/min, respectively. Both BYK and G-336 dispersants showed an increase in the polishing rate of over 50% compared with the initial rate of the Base condition. The application of defoaming polymers led to an increase in polishing efficiency. Additionally, the nitride-stopping efficiency was measured in the order of G-336, Depol, and BYK for defoaming polymer-applied ceria slurries. Specifically, the ceria slurry with G-336 defoaming polymer exhibited significantly higher selectivity compared with the other samples. Particularly, the selectivity ratio for the ceria slurry with the G-336 dispersant was confirmed to be 80. This suggests that the incorporation of the G-336 defoaming polymer into the dispersant polymer leads to superior thermal stability and increasing uniformity, as evidenced by the absence of bubble generation, as observed in Figure 12.

## 4. Conclusions

In this study, research was conducted on calcined ceria nanoparticles using SMA-1000 dispersant polymer applied with three types of defoaming polymers as additives, aiming to eliminate bubbles, enhance particle stability, and increase the polishing selection ratio. The calcined cerium oxide (CeO_2_) exhibited sizes ranging from tens to hundreds of nanometers, revealing a cerium content of 74.3% in EDX analysis. Stability tests were conducted on the SMA-1000 dispersant polymer at concentrations of 4.0%, 4.5%, and 5.0% at 60 °C for 60 days. As the concentration increased, the pH slightly increased, while the viscosity showed a slight change. On the other hand, the conductivity increased to 231 μS/cm, 248 μS/cm, and 262 μS/cm, and the size also exhibited an increasing trend at 216 nm, 224 nm, and 232 nm, respectively.

The stability tests were performed on ceria slurries containing SMA dispersant polymer and three types of defoaming polymers for pH, conductivity, viscosity, and size analysis. The results indicated that the addition of the G-336 defoaming polymer to the ceria slurry resulted in the following high conductivity values: 218 μS/cm at a concentration of 0.01%, 219 μS/cm at a concentration of 0.025%, and 245 μS/cm at a concentration of 0.050%. The DLS results also revealed smaller sizes, measuring 179 nm at concentrations of 0.010% and 0.025%, and 180 nm at a concentration of 0.050%, when compared with the other defoaming polymers. This suggests that the G-336 defoaming polymer has a positive effect on particle behavior in ceria nanoparticles containing SMA-1000 dispersant polymer, providing excellent colloidal stability.

Furthermore, AF4 analysis was performed to confirm the size distribution, and the results showed a monodisperse fractogram for ceria slurries with the BYK and G-336 defoaming polymers compared with defoaming polymers.

Finally, polishing rate tests were conducted on ceria slurries with defoaming polymers. The results showed superior polishing efficiency for the ceria slurry containing the G-336 defoaming polymer and SMA-1000 dispersant polymer. The PETEOS polishing rate was 5417 Å/min and the nitride polishing rate was 68 Å/min, with a high selection ratio of 80. This indicates excellent polishing efficiency when applying the G-336 defoaming polymer, suggesting that it did not cause bubble formation and positively influenced particle behavior during dispersion.

In conclusion, the enhanced CMP performance obtained using the G-336 defoaming polymer and SMA-1000 dispersant polymer developed in this study suggests potential innovative advancements in slurry manufacturing and process efficiency for future CMP applications.

## Figures and Tables

**Table 1:** Weight and atom concentration of each element (relative %) obtained from SEM-EDX results for cerium carbonate and cerium oxide after calcined at 800 °C.

Element	Cerium Carbonate		Cerium Oxide	
	wt%	Atomic %	wt%	Atomic %
C	10.8	25.2	5.46	20.2
O	36.8	64.3	20.2	56.2
Ce	52.4	10.5	74.3	23.5

**Table 2:** The pH and molecular weight (MW) of the synthesized SMA dispersant polymer.

Name	pH	M_w_
SMA 1000	8.07	8.60 × 10^4^

**Table 3:** The storage stability of the ceria slurry according to the SMA-1000 concentration.

Conc. (%)	pH	Conductivity (ms/cm)	Viscosity (cP)	DLS (nm)
4.0	9.43 ± 0.0300	231 ± 30.5	1.34 ± 0.0300	216 ± 22.3
4.5	9.45 ± 0.0400	248 ± 35.6	1.34 ± 0.0200	224 ± 27.6
5.0	9.46 ± 0.0400	262 ± 38.2	1.35 ± 0.0200	232 ± 18.3

All evaluations were measured three times, and the average value was determined.

**Table 4:** The storage stability of the ceria slurry according to Depol, BYK, G-336 defoaming polymers at various concentrations.

Defoaming Polymer	Conc (%)	pH	Conductivity (μs/cm)	Viscosity (cP)	DLS (nm)
Depol	0.010	9.68 ± 0.0500	205 ± 10.9	1.33 ± 0.0100	183 ± 9.16
	0.025	9.68 ± 0.0600	208 ± 13.0	1.32 ± 0.0100	183 ± 9.82
	0.050	9.67 ± 0.0500	209 ± 14.9	1.32 ± 0.0100	181 ± 4.28
BYK	0.010	9.68 ± 0.0500	206 ± 11.1	1.32 ± 0.0100	183 ± 7.77
	0.025	9.68 ± 0.0600	209 ± 11.2	1.31 ± 0.0100	184 ± 7.40
	0.050	9.68 ± 0.0600	212 ± 15.0	1.31 ± 0.0100	197 ± 4.80
G-336	0.010	9.64 ± 0.0200	218 ± 9.07	1.32 ± 0.0100	179 ± 6.43
	0.025	9.65 ± 0.0300	219 ± 8.93	1.32 ± 0.0100	179 ± 4.75
	0.050	9.66 ± 0.0300	245 ± 11.6	1.32 ± 0.0100	180 ± 4.32

All evaluations were measured three times, and the average value was determined.

**Table 5:** Particle size distribution (PSD) of the ceria slurry by DLS analysis.

Size Distribution (nm)	Base	Depol	BYK	G-336
Volume	117 ± 60.8	128 ± 57.1	91.4 ± 45.6	103 ± 47.9
Number	83.2 ± 23.2	92.6 ± 26.0	64.8 ± 18.5	74.3 ± 21.1

All evaluations were measured three times, and the average value was determined.

**Table 6:** Particle size analysis (PSD) of the ceria slurry by AF4 separation.

Size Distribution (nm)	Base	Depol	BYK	G-336
Main	72.2 ± 1.34	62.6 ± 3.20	73.3 ± 1.39	82.0 ± 7.69
Minor	283 ± 3.01	293 ± 1.52	N.D	N.D

All evaluations were measured three times, and the average value was determined. N.D: not detected.

**Table 7:** CMP efficiency results of ceria slurry applied with 3 types of defoaming polymers on the PETEOS and nitride surface.

Polishing Rate (Å/min)	Base	Depol	BYK	G-336
PETEOS	3493	4650	5558	5417
Nitride	60	75	83	68
Selection ratio	59	62	67	80

## Data Availability

Data are contained within the article.

## References

[1] Nanz G., Camilletti L.E. (1995). Modeling of chemical-mechanical polishing: A review. Semicond. Manuf..

[2] Bouzid D., Belkhie N., Aliouane T. (2012). Optical glass surfaces polishing by cerium oxide particles. IOP Conf. Ser. Mater. Sci. Eng..

[3] Wang L., Zhang K., Song Z., Feng S. (2007). Ceria concentration effect on chemical mechanical polishing of optical glass. Appl. Surf. Sci..

[4] Zhong Z.W., Tian Y.B., Ng J.H., Ang Y.J. (2014). Chemical mechanical polishing (CMP) processes for manufacturing optical silicon substrates with shortened polishing time. Mater. Manuf. Process..

[5] Cheng J., Huang S., Li Y., Wang T., Xie L., Lu X. (2020). RE (La, Nd and Yb) doped CeO_2_ abrasive particles for chemical mechanical polishing of dielectric materials: Experimental and computational analysis. Appl. Surf. Sci..

[6] Oh S., Seok J. (2009). An integrated material removal model for silicon dioxide layers in chemical mechanical polishing processes. Wear.

[7] Wang Y., Zhao Y.W., Chen X. (2012). Chemical mechanical planarization from macroscale to molecular-scale. Mater. Manuf. Process..

[8] Tian Y.B., Ang Y.J., Zhong Z.W., Xu H., Tan R. (2013). Chemical mechanical polishing of glass disk substrates: Preliminary experimental investigation. Mater. Manuf. Process..

[9] Xu G., Zhang Z., Meng F., Liu L., Liu D., Shi C., Cui X., Wang J., Wen W. (2023). Atomic-scale surface of fused silica induced by chemical mechanical polishing with controlled size spherical ceria abrasives. J. Manuf. Process..

[10] Kim J.Y., Han S.J., Kim S.S. (2010). The Enhanced Electrophoresis Method in Leachate System for Repairing of Leaks in Waste Landfill Geomembrane Liner. J. Korean Soc. Civ. Eng..

[11] Song G.D., Kim M.H., Lee Y.T., Maeng W.Y. (2013). Improvement in the Dispersion Stability of Iron Oxide (Magnetite, Fe_3_O_4_) Particles with Polymer Dispersant Injection. Appl. Chem. Eng..

[12] Lee S.B., Park H.H., Bae I.S., Yoon J.S., Kim B.J. (2002). Effect of Al, Al_2_O_3_ Dispersants and Heat Treatment on Deposits from Watt’s Ni Plating Bamth. Korean J. Mater. Res..

[13] Hedrick J.B., Sinha S.P. (1994). Cerium-based polishing compounds: Discovery to manufacture. J. Alloys Compd..

[14] Urie R.W., Wylie A.W. (1947). Rare earth oxides for glass polishing. J. Soc. Chem. Ind..

[15] Kosynkin V.D., Arzgatkina A.A., Ivanov E.N., Chtoutsa M.G., Grabko A.I., Kardapolov A.V. (2000). The study of process production of polishing powder based on cerium dioxide. J. Alloys Compd..

[16] Kim D.-H., Kim S.-K., Kang H.-G., Park J.-G., Paik U. (2005). The effect of cerium precursor agglomeration on the synthesis of ceria particles and its influence on shallow trench isolation chemical mechanical polishing performance. Jpn. J. Appl. Phys..

[17] Li Y., Cheng C., Chen W., Hu J., Zhou X., Hu P. (2006). Preparation and polishing property of ultra-fine ceria by calcining hydrate cerium acetate directly. Chin. J. Inorg. Chem..

[18] Kurokawa S., Toyama T., Hayashi T., Suda E., Tokuda J. Controllable CMP of oxide flim by using colloidal ceria slurry. Proceedings of the ICPT 2017—International Conference on Planarization/CMP Technology.

[19] Kim N.Y., Kim G., Sun H., Hwang U., Kim J., Kwak D., Park I.-K., Kim T., Suhr J., Nam J.-D. (2022). A nanoclustered ceria abrasives with low crystallinity and high Ce^3+^/Ce^4+^ ratio for scratch reduction and high oxide removal rates in the chemical mechanical planarization. J. Mater. Sci..

[20] Kim E., Hong J., Seok H., Kim T. (2022). Photo oxidative degradation of polyacids derived ceria nanoparticle modulation for chemical mechanical polishing. Sci. Rep..

[21] Sahir S., Yerribonia N.P., Han S.Y., Han K.M., Kim T.G., Mahadev N., Park J.G. (2021). Investigation of the effect of different cleaning forces on Ce-O-Si bonding during oxide post-CMP cleaning. Appl. Surf. Sci..

[22] Myong K.K., Byun J., Choo M.J., Kim H., Kim J., Lim Y.T., Kim J.J. (2021). Direct and quantitative study of ceria–SiO_2_ interaction depending on Ce^3+^ concentration for chemical mechanical planarization (CMP) cleaning. Mater. Sci. Semicond. Process..

[23] Kim K., Yi D.K., Paik U. (2017). Increase in Ce^3+^ concentration of ceria nanoparticles for high removal rate of SiO_2_ in chemical mechanical planarization. ECS J. Solid State Sci. Technol..

[24] Netzband C.M., Dunn K. (2019). Investigation into the effect of CMP slurry chemicals on ceria abrasive oxidation state using XPS. ECS J. Solid State Sci. Technol..

[25] Lin S.-S. (2004). Preparing an active cerium oxide catalyst for the catalytic incineration of aromatic hydrocarbons. Appl. Catal..

[26] He H.-W., Wu X.-Q., Ren W., Peng S., Xi Y., Zhi S.T. (2012). Synthesis of crystalline cerium dioxide hydrosol by a sol–gel method. Ceram. Int..

[27] Chen H.I., Chang H.-Y. (2005). Synthesis of nanocrystalline cerium oxide particles by the precipitation method. Ceram. Int..

[28] Rojas S., Gispert J.D., Abad S., Buaki-Sogo M., Victor V.M. (2012). In Vivo biodistribution of amino-functionalized ceria nanoparticles in rats using positron emission tomography. Mol. Pharm..

[29] Annis J.W., Fisher J.M., Thompsett D., Walton R.I. (2021). Solvothermal synthesis routes to substituted cerium dioxide materials. Inorganics.

[30] Ali M.M., Mahdi H.S., Parveen A. (2018). Optical properties of cerium oxide (CeO_2_) nanoparticles synthesized by hydroxide mediated method. AIP Conf. Proc..

[31] Wakamatsu K., Kurokawa S., Toyama T., Hayashi T. (2019). CMP characteristics of quarts glass substrate by aggregated colloidal ceria slurry. Precis. Eng..

[32] Lyklema J., van Leeuwen H.P., Minor M. (1999). DLVO-theory, a dynamic re-interpretation. Adv. Colloid Interface Sci..

[33] Kabir H., Garg N. (2023). Rapid prediction of cementitious initial sorptivity via surface wettability. NPJ Mater. Degrad..

[34] Deltombe R., Kubiak K.J., Bigerelle M. (2014). How to Select the Most Relevant 3D Roughness Parameters of a Surface. Scanning.

[35] Seo J., Lee J.W., Moon J., Sigmund W., Paik U. (2014). Role of the surface chemistry of ceria surfaces on silicate adsorption. ACS Appl. Mater. Interfaces.

[36] Kim S.K., Lee S., Paik U., Katoh T., Park J.G. (2003). Influence of the electrokinetic behaviors of abrasive ceria particles and the deposited plasma-enhanced tetraethylorthosilicate and chemically vapor deposited Si_3_N_4_ films in an aqueous medium on chemical mechanical planarization for shallow trench isolation. J. Mater. Res..

[37] Hackley V.A. (1997). Colloidal processing of silicon nitride with poly (acrylic acid): I, adsorption and electrostatic interactions. J. Am. Ceram. Soc..

[38] Sehgal A., Lalatonne Y., Berret J.-F., Morvan M. (2005). Precipitation−redispersion of cerium oxide nanoparticles with poly(acrylic acid): Toward stable dispersions. Langmuir.

[39] Lee J., Bae J., Kim W., Lee S. (2022). A Study on Aqueous Dispersing of Carbon Black Nanoparticles Surface-Coated with Styrene Maleic Acid (SMA) Copolymer. Polymers.

